# Supplementary material for: Claudin-11 regulates immunological barrier formation and spermatogonial proliferation through stem cell factor
Source: Commun Biol. 2025 Jan 30;8:148. doi: 10.1038/s42003-025-07592-0 (PMC11782696; doi:10.1038/s42003-025-07592-0)
Supplement: Supplementary file 2 — Supplementary Information [file 42003_2025_7592_MOESM2_ESM.pdf]

# Supplementary information

**Claudin-11 regulates immunological barrier formation and spermatogonial proliferation through stem cell factor**

Taichi Sugawara[1,*], Kayoko Sonoda[1], Nattapran Chompusri[1], Kazuhiro Noguchi[1], Seiji Okada[2], Mikio Furuse[3,4,5], and Tomohiko Wakayama[1]

[1]Department of Histology, Graduate School of Medical Sciences, Kumamoto University, Kumamoto, Japan

[2]Division of Hematopoiesis, Joint Research Center for Human Retrovirus Infection, Kumamoto University, Kumamoto, Japan

[3]Division of Cell Structure, National Institute for Physiological Sciences, National Institute of Natural Sciences, Okazaki, Aichi, Japan

[4]Physiological Sciences Program, Graduate Institute for Advanced Studies, SOKENDAI, Okazaki, Aichi, Japan

[5]Nagoya University Graduate School of Medicine, Nagoya, Japan

[*]Corresponding author: Taichi Sugawara

Department of Histology, Graduate School of Medical Sciences, Kumamoto University, 1-1-1 Honjo, Chuo-ku, Kumamoto, 860-8556, Japan

Tel.: +81-96-373-5047, Fax: +81-96-373-5048, E-mail: tsugawara@kumamoto-u.ac.jp

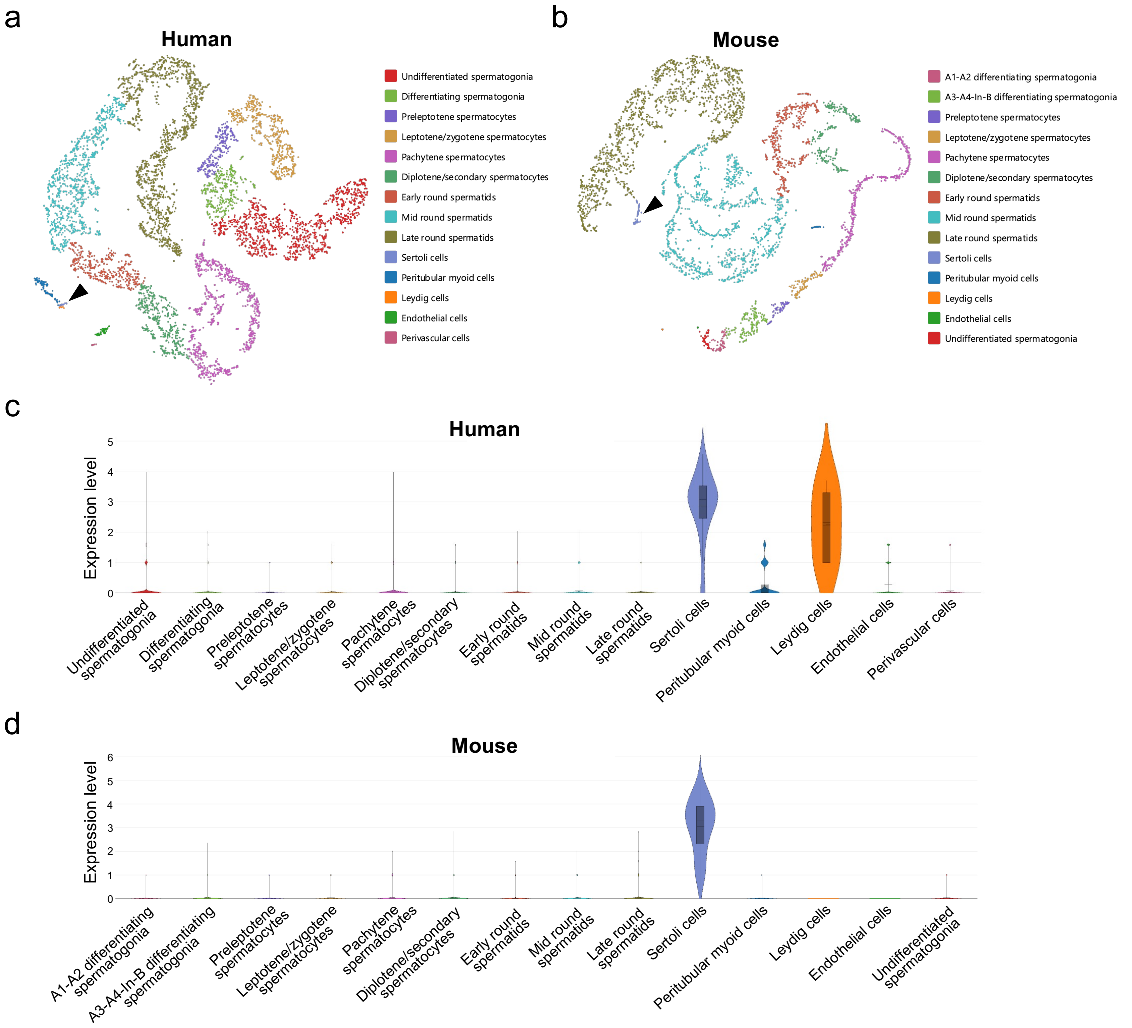


**Supplementary Fig. 1 | mRNA expression of *CLDN11*/*Cldn11* in human and mouse testicular cells.**

**a**, **b** t-Distributed stochastic neighbor embedding (t-SNE) plots showing 10x Genomics profiling of unselected spermatogenic cells from human testes (**a**) and mouse testes (**b**). Cell clusters were identified by expression of key marker genes. Black arrowheads indicate clusters of Sertoli cells. **c**, **d** Violin plots showing mRNA expression levels of human *CLDN11* (**c**) and mouse *Cldn11* (**d**) among the clusters shown in (**a**) and (**b**), respectively. Based on published single-cell RNA-seq data[33], t-SNE plots (**a**, **b**) and violin plots (**c**, **d**) were shown.

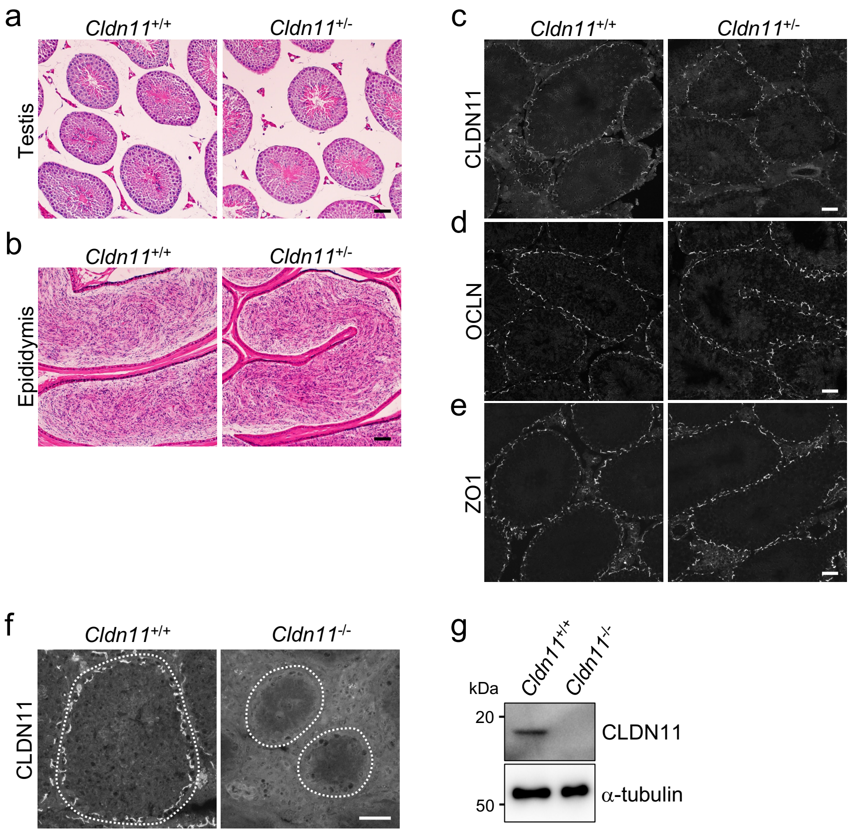


**Supplementary Fig. 2 | Histological, immunohistochemical, and western blotting analyses using *Cldn11*$^{+/+}$, *Cldn11*$^{+/-}$, and *Cldn11*$^{-/-}$ mice.**

**a**, **b** Hematoxylin and eosin staining of sections prepared from testes (**a**) and the cauda epididymides (**b**) of *Cldn11*$^{+/+}$ and *Cldn11*$^{+/-}$ mice. **c**–**e** Immunohistochemistry of frozen testis sections from *Cldn11*$^{+/+}$ and *Cldn11*$^{+/-}$ mice using anti-CLDN11 (**c**), anti-OCLN (**d**), or anti-ZO1 antibody (**e**). **f** Immunohistochemistry of frozen testis sections from *Cldn11*$^{+/+}$ and *Cldn11*$^{-/-}$ mice using anti-CLDN11 antibody. White dotted lines outline the seminiferous tubules. **g** Western blotting of testis lysates prepared from *Cldn11*$^{+/+}$ and *Cldn11*$^{-/-}$ mice using anti-CLDN11 and anti-α-tubulin antibodies. Scale bars: 50 μm (**a**–**f**).

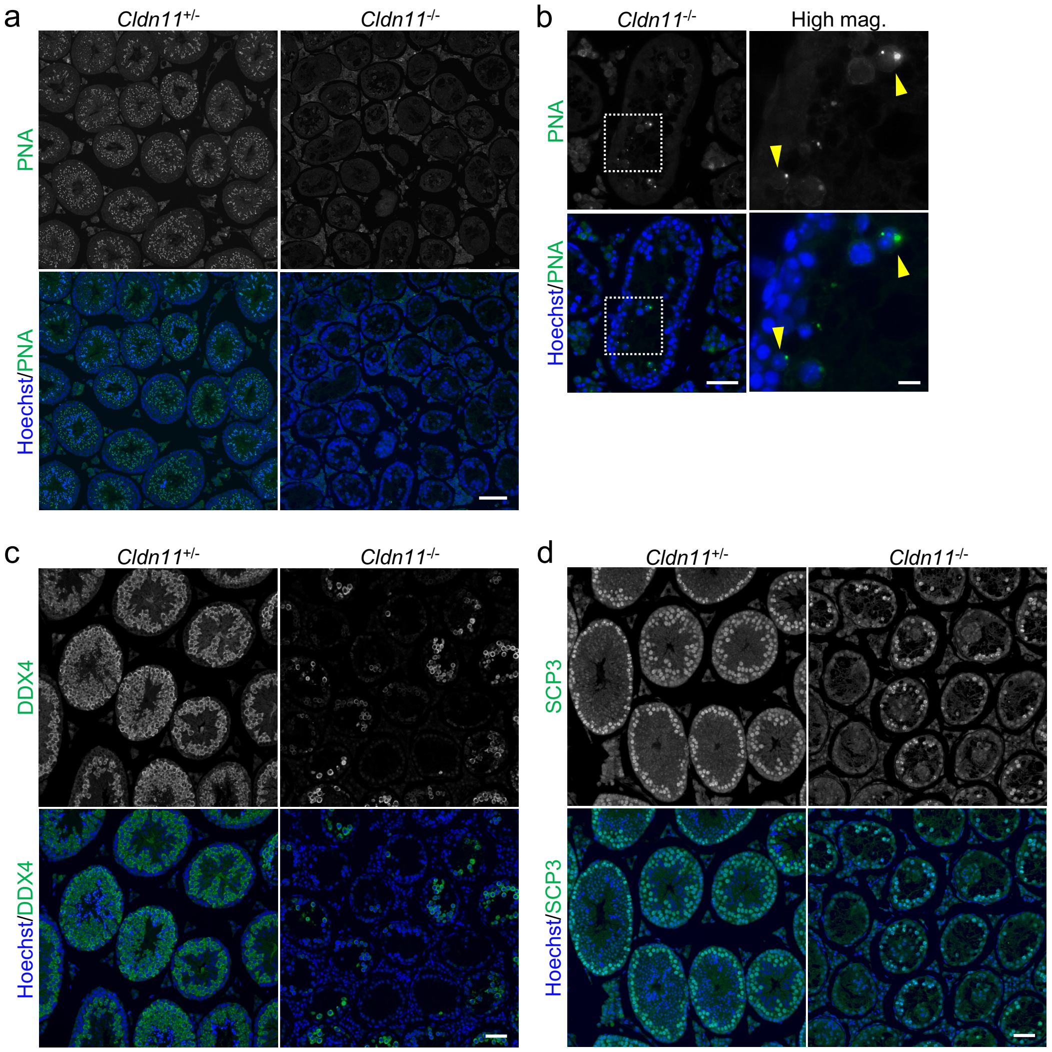


**Supplementary Fig. 3 | The number of PNA lectin$^{+}$, DDX4$^{+}$, and SCP3$^{+}$ cells is decreased in *Cldn11$^{-/-}$* mouse testes.**

**a**, **b** Testis sections from *Cldn11$^{+/-}$* and *Cldn11$^{-/-}$* mice (**a**), or *Cldn11$^{-/-}$* mice (**b**) were stained using PNA lectin. PNA lectin$^{+}$ cells were occasionally observed, as shown by yellow arrowheads (**b**). White dotted squares indicate the regions shown in high magnification images (**b**, High mag.). **c**, **d** Immunohistochemistry of testis sections prepared from *Cldn11$^{+/-}$* and *Cldn11$^{-/-}$* mice using anti-DDX4 (**c**) or anti-SCP3 antibody (**d**). Scale bars: 100 μm (**a**), 50 μm (**b**–**d**), and 10 μm (**b**, High mag.).

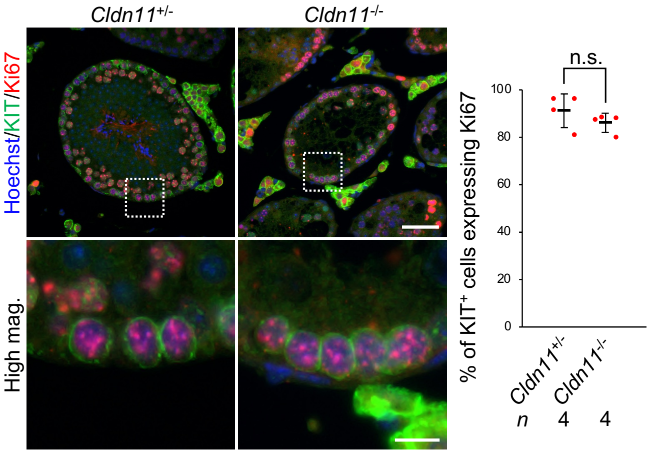


**Supplementary Fig. 4 | Residual differentiating spermatogonia in *Cldn11*$^{-/-}$ mice maintain proliferative capacity.**

Immunohistochemistry of testis sections from *Cldn11*$^{+/-}$ and *Cldn11*$^{-/-}$ mice using anti-KIT and anti-Ki67 antibodies. White dotted squares indicate the regions shown in high magnification images (High mag.). A total of 516 or 497 KIT$^{+}$ cells on seminiferous tubule sections from testes of four biologically independent *Cldn11*$^{+/-}$ or *Cldn11*$^{-/-}$ mice, respectively, were analyzed. The proportion of KIT$^{+}$ cells expressing Ki67 is shown in the graph. Red dots indicate biological replicates of mice. Data are shown as mean ± SD and were analyzed by Student's *t*-test. n.s. (not significant): $P > 0.05$. Scale bars: 50 μm and 10 μm (High mag.).

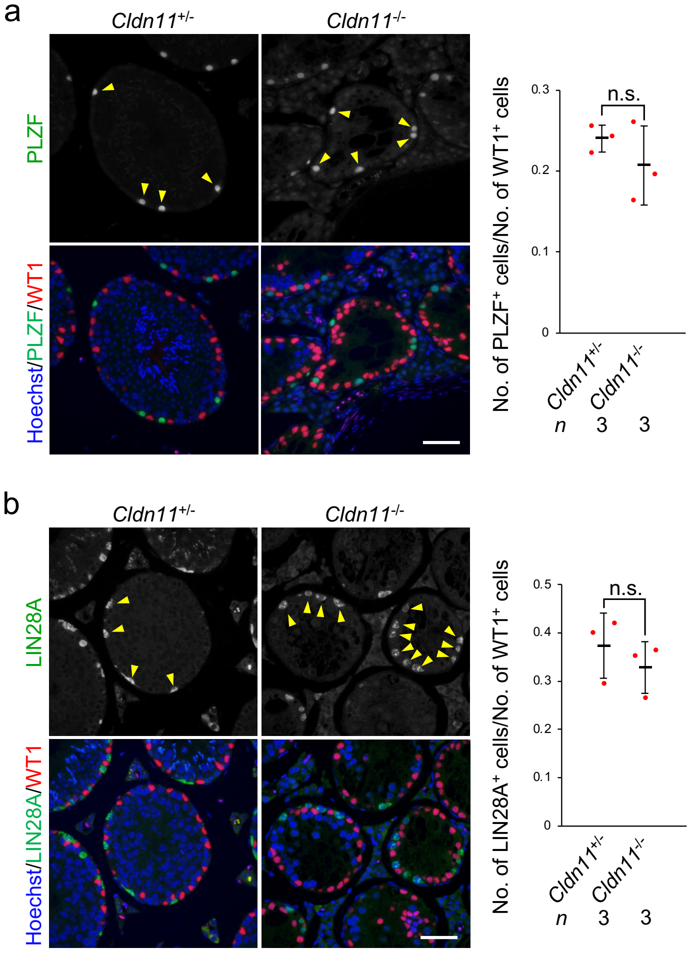


**Supplementary Fig. 5 | CLDN11 is dispensable for maintenance of undifferentiated spermatogonia.**

**a**, **b** Immunohistochemistry of testis sections from *Cldn11*$^{+/-}$ and *Cldn11*$^{-/-}$ mice using anti-PLZF and anti-WT1 (**a**) or anti-LIN28A and anti-WT1 antibodies (**b**). Cells expressing PLZF (**a**) or LIN28A (**b**) in seminiferous tubules are shown by yellow arrowheads. The number of PLZF$^{+}$ (**a**) or LIN28A$^{+}$ (**b**) cells on seminiferous tubule sections from testes of *Cldn11*$^{+/-}$ and *Cldn11*$^{-/-}$ mice was normalized to the number of WT1$^{+}$ Sertoli cells. A total of 195–226 seminiferous tubules on testis sections from three biologically independent *Cldn11*$^{+/-}$ or *Cldn11*$^{-/-}$ mice were analyzed. Red dots indicate biological replicates of mice. Data are shown as mean ± SD and were analyzed by

Student's *t*-test. n.s. (not significant): $P > 0.05$. Scale bars: 50 μm (**a**, **b**).

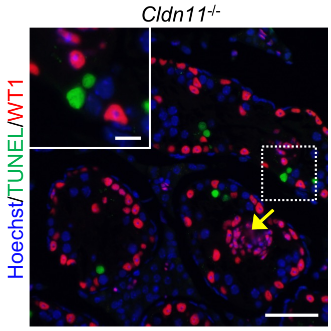


**Supplementary Fig. 6 | TUNEL assay with immunohistochemistry for WT1.**

TUNEL assay with immunohistochemistry of testis sections prepared from *Cldn11*$^{-/-}$ mice using anti-WT1 antibody. The inset shows a magnified image of the region indicated by a white dotted square. A yellow arrow shows a TUNEL$^{-}$ Sertoli cell cluster. Scale bars: 50 µm and 10 µm (inset).

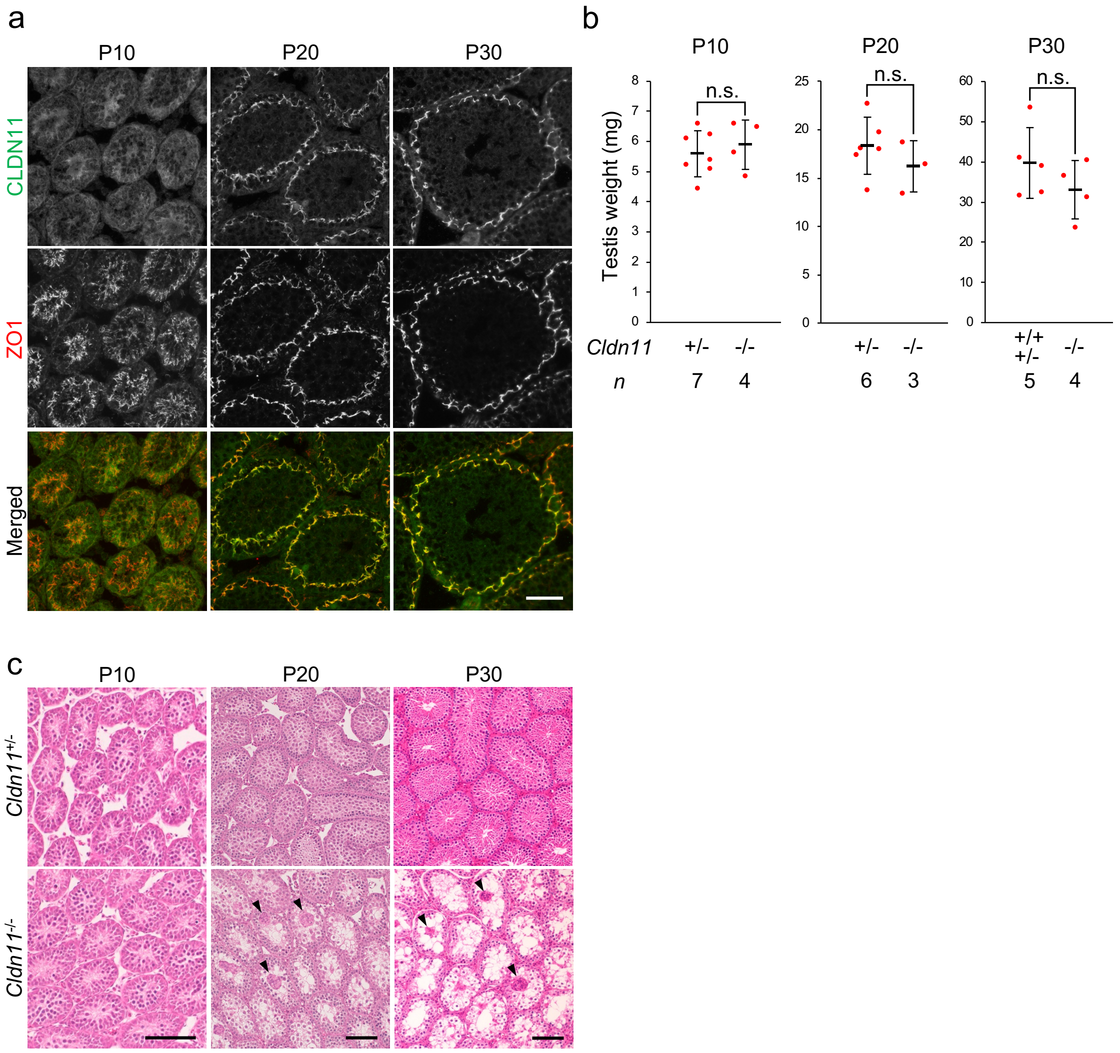


**Supplementary Fig. 7 | CLDN11 is necessary to accomplish the first wave of spermatogenesis, but is dispensable for its onset.**

**a** Immunohistochemistry of frozen testis sections from P10, P20, and P30 wild-type mice using anti-CLDN11 and anti-ZO1 antibodies. **b** Testis weight of P10, P20, and P30 mice from each genotype shown in the graph. The number of biologically independent mice in each group is shown as *n* in the graph. Red dots indicate biological replicates of mice. Data are shown as mean ± SD and were analyzed by Student's *t*-test. n.s. (not significant): $P > 0.05$. **c** Hematoxylin and eosin staining of testis sections from *Cldn11*$^{+/-}$ and *Cldn11*$^{-/-}$ mice at P10, P20, and P30. Black arrowheads indicate Sertoli cell clusters. Scale bars:

50 μm (**a**) and 100 μm (**c**).

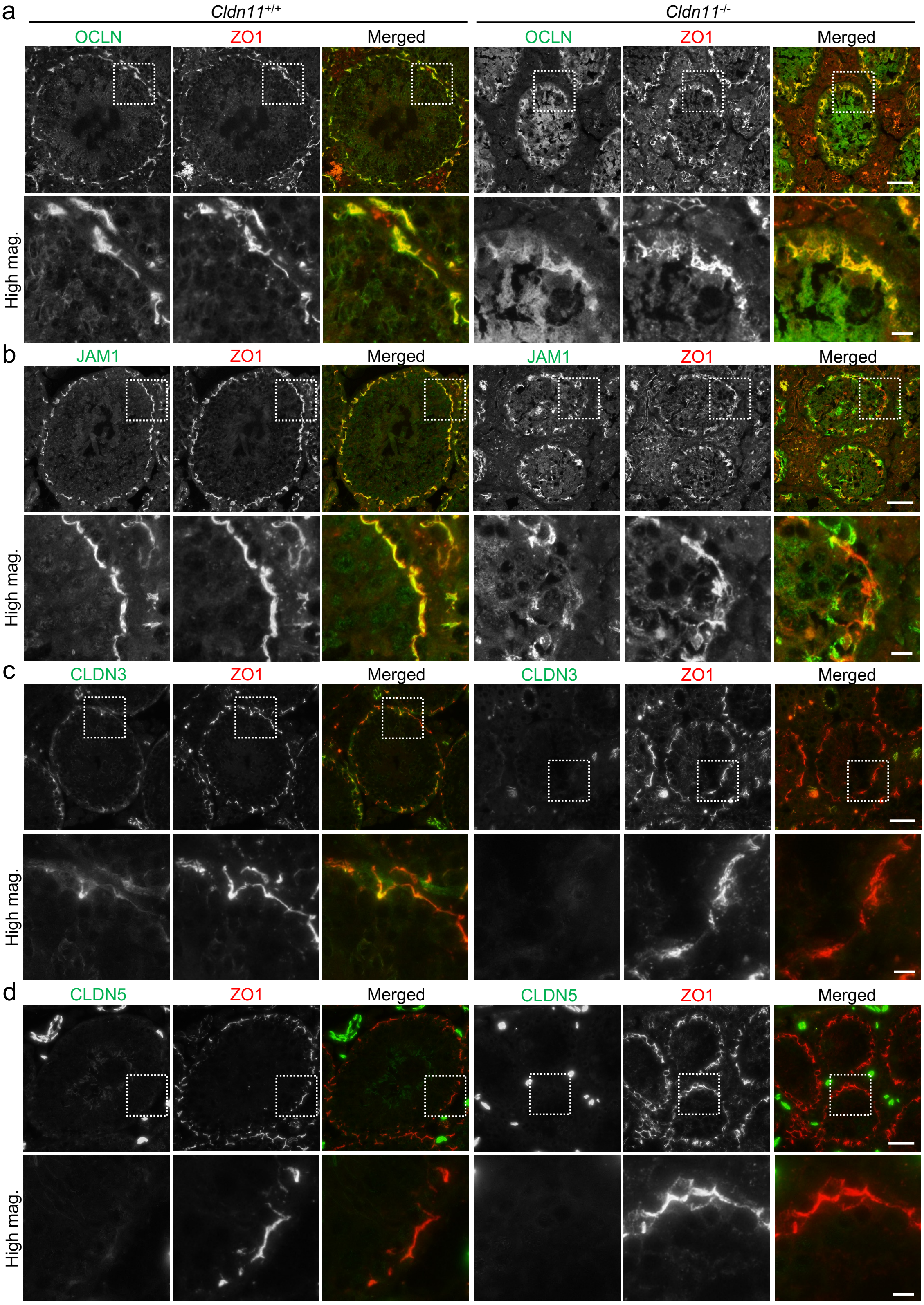


**Supplementary Fig. 8 | Localization of TJ-associated proteins in seminiferous tubules of *Cldn11*$^{-/-}$ mice.**

**a**–**d** Immunohistochemistry of frozen testis sections from *Cldn11*$^{+/+}$ and *Cldn11*$^{-/-}$ mice using anti-OCLN and anti-ZO1 (**a**), anti-JAM1 and anti-ZO1 (**b**), anti-CLDN3 and anti-ZO1 (**c**), or anti-CLDN5 and anti-ZO1 antibodies (**d**). White dotted squares indicate the regions shown in high magnification images (High mag.). Scale bars: 50 μm (**a**–**d**) and 10 μm (**a**–**d**, High mag.).

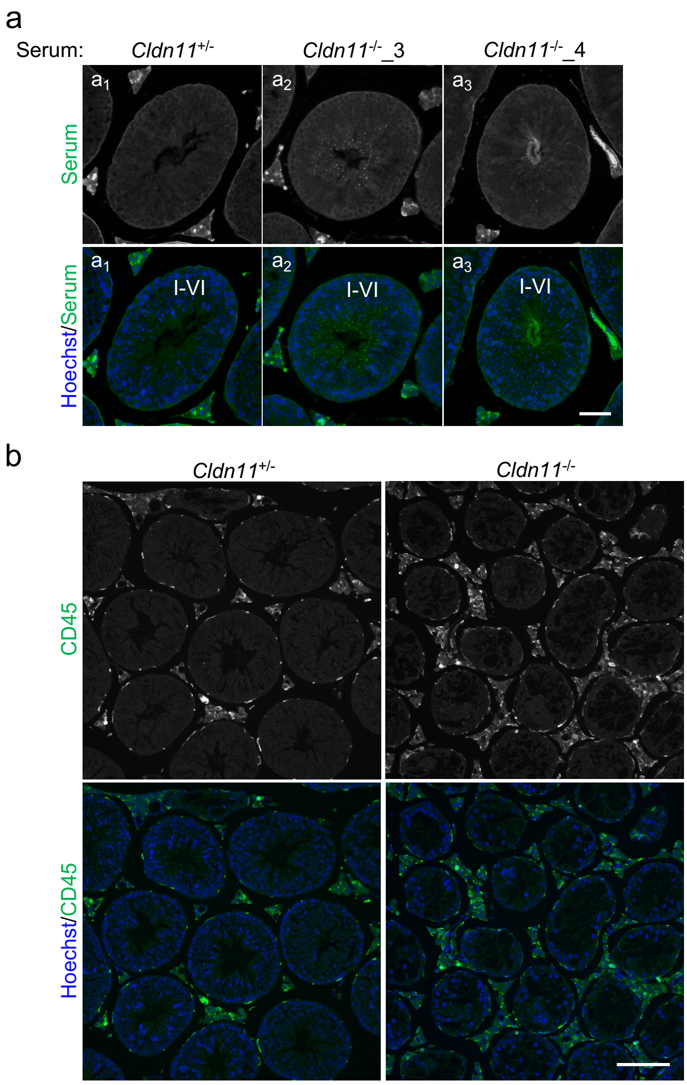


**Supplementary Fig. 9 | Production of autoantibodies against antigens of spermatogenic cells in *Cldn11*$^{-/-}$ mice and distribution of leukocytes in *Cldn11*$^{-/-}$ mouse testes.**

**a** Immunohistochemistry of wild-type testis sections using sera from a *Cldn11*$^{+/-}$ mouse (**$a_1$**) and *Cldn11*$^{-/-}$ mice (**$a_2$** and **$a_3$**) as primary antibodies. Dot-like signals (**$a_2$**) or signals derived from flagella of elongated spermatids (**$a_3$**) were detected at the adluminal compartment of seminiferous tubules. Stages of spermatogenesis are shown by Roman numerals. **b** Immunohistochemistry of testis sections from *Cldn11*$^{+/-}$ and *Cldn11*$^{-/-}$ mice using anti-CD45 antibody. Scale bars: 50 μm (**a**) and 100 μm (**b**).

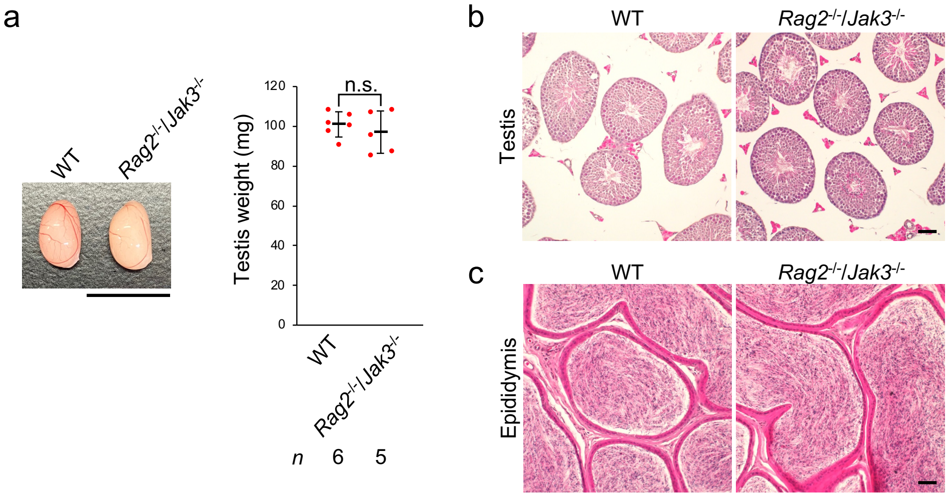


**Supplementary Fig. 10 | Normal spermatogenesis in *Rag2*$^{-/-}$/*Jak3*$^{-/-}$ mice.**

**a** Appearance and weight of testes from wild-type (WT) and *Rag2*$^{-/-}$/*Jak3*$^{-/-}$ mice. The number of biologically independent mice in each group is shown as *n* in the graph. Red dots indicate biological replicates of mice. Data are shown as mean ± SD and were analyzed by Student's *t*-test. n.s. (not significant): $P > 0.05$. **b**, **c** Hematoxylin and eosin staining of sections from testes (**b**) and the cauda epididymides (**c**) of WT and *Rag2*$^{-/-}$/*Jak3*$^{-/-}$ mice. Scale bars: 1 cm (**a**) and 50 µm (**b**, **c**).

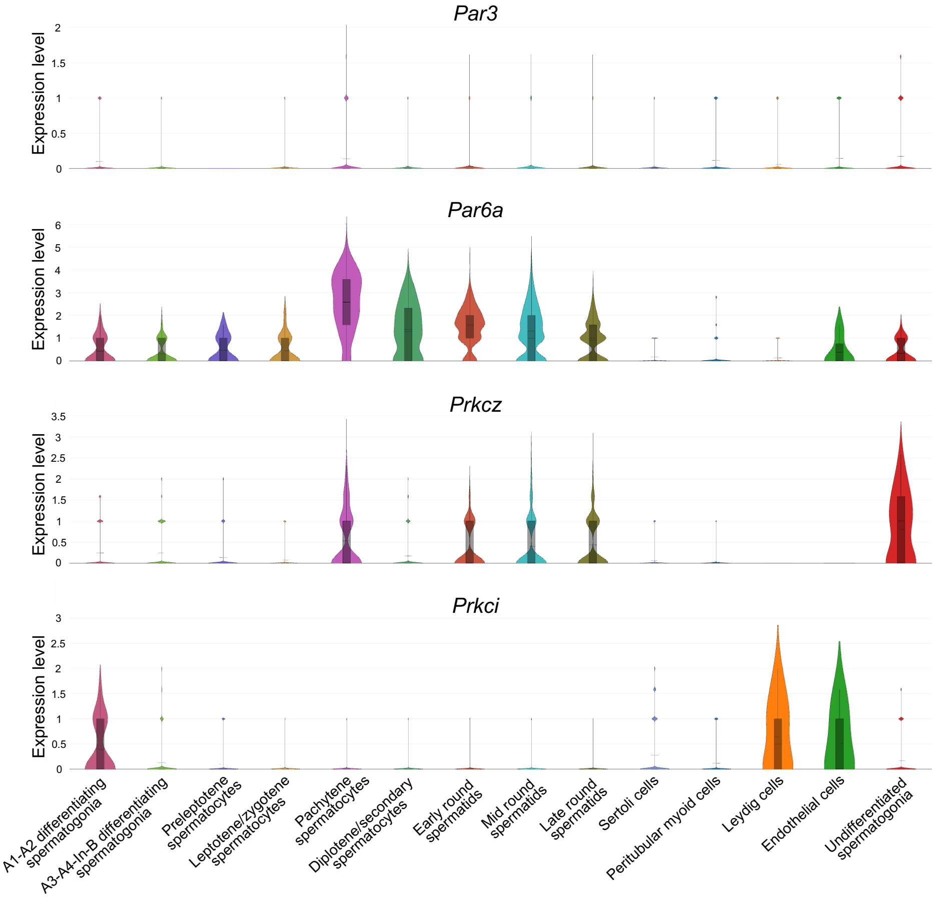


**Supplementary Fig. 11 | mRNA expression of *Par3*, *Par6a*, *Prkcz*, and *Prkci* in mouse testicular cells.**

Violin plots showing mRNA expression levels of mouse *Par3*, *Par6a*, *Prkcz*, and *Prkci* among the clusters shown in Supplementary Fig. 1b.

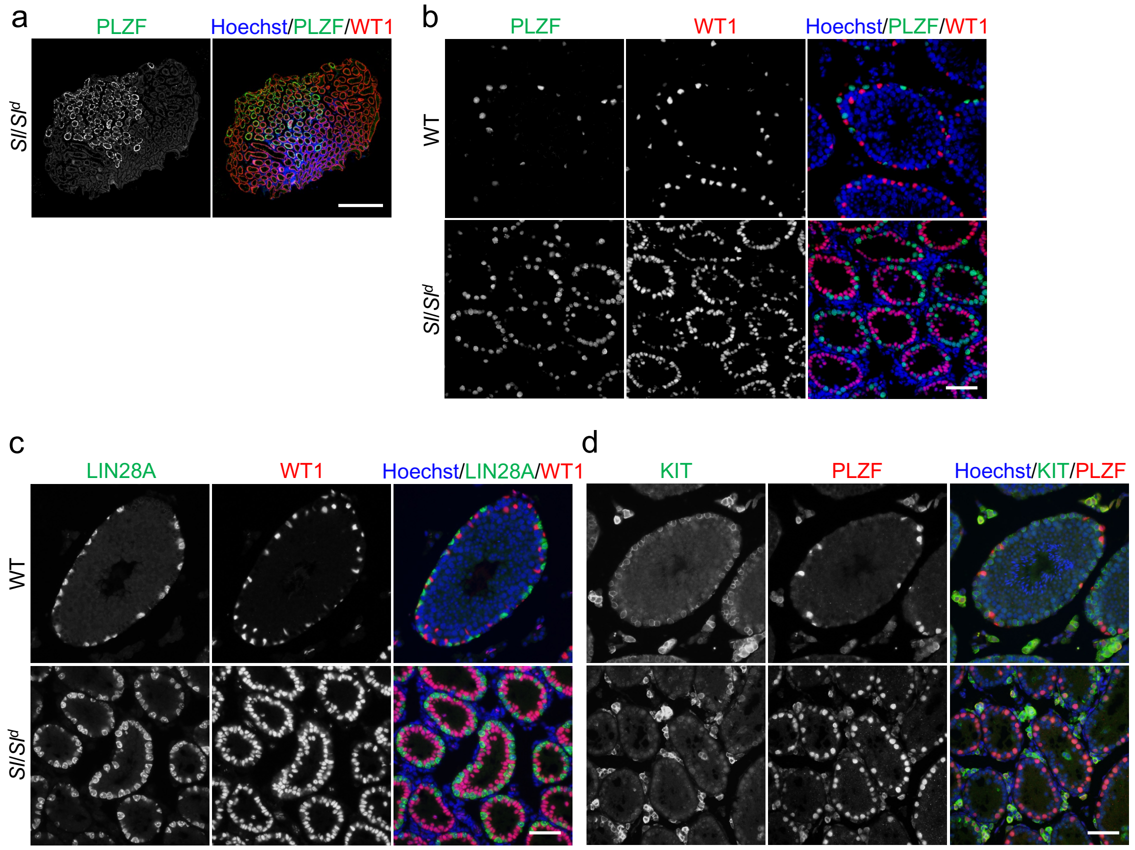


**Supplementary Fig. 12 | Undifferentiated spermatogonia, but not differentiating spermatogonia, are maintained in some seminiferous tubules of $Sl/Sl^{d}$ mice.**

**a** Immunohistochemistry of testis sections from $Sl/Sl^{d}$ mice at 6 weeks of age using anti-PLZF and anti-WT1 antibodies. PLZF$^{+}$ cells were observed in some seminiferous tubules. **b–d** Immunohistochemistry of testis sections from wild-type (WT) and $Sl/Sl^{d}$ mice at 6 weeks of age using anti-PLZF and anti-WT1 (**b**), anti-LIN28A and anti-WT1 (**c**), or anti-KIT and anti-PLZF antibodies (**d**). Scale bars: 500 μm (**a**) and 50 μm (**b–d**).

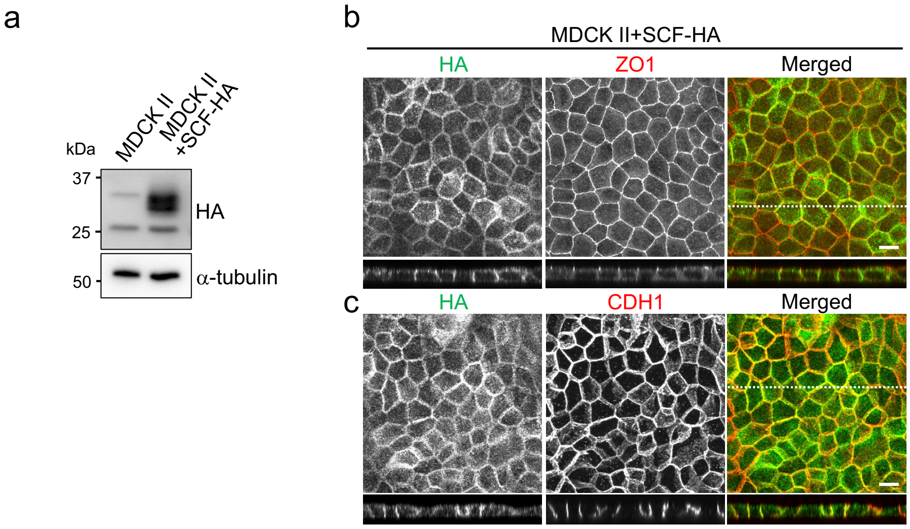


**Supplementary Fig. 13 | SCF-HA is localized to the lateral membrane in MDCK II cells.**

**a** SCF tagged with HA at its C-terminus (SCF-HA) was stably expressed in MDCK II cells. Western blotting of lysates prepared from parental MDCK II cells and MDCK II cells stably expressing SCF-HA using anti-HA and anti-α-tubulin antibodies. **b**, **c** Immunofluorescence staining of MDCK II cells stably expressing SCF-HA using anti-HA and anti-ZO1 (**b**), or anti-HA and anti-CDH1 antibodies (**c**). Maximum intensity projections of Z-stacked images (top) and the corresponding Z-stacked images along white dotted lines (bottom) are shown. Scale bars: 10 μm (**b**, **c**).

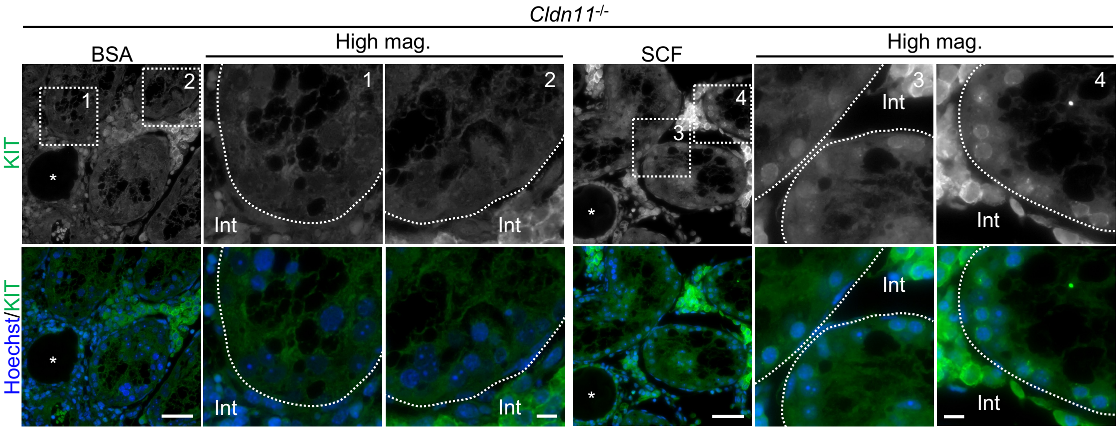


**Supplementary Fig. 14 | Differentiating spermatogonia are observed in seminiferous tubules close to SCF-soaked beads transplanted into the testicular interstitium in *Cldn11*$^{-/-}$ mice.**

Immunohistochemistry using anti-KIT antibody on sections from *Cldn11*$^{-/-}$ mouse testes, with BSA- or SCF-soaked beads transplanted into the interstitium (Int). White dotted squares indicate the regions shown in high magnification images (High mag.). White dotted lines outline seminiferous tubules. Asterisks indicate beads. Scale bars: 50 μm and 10 μm (High mag.)

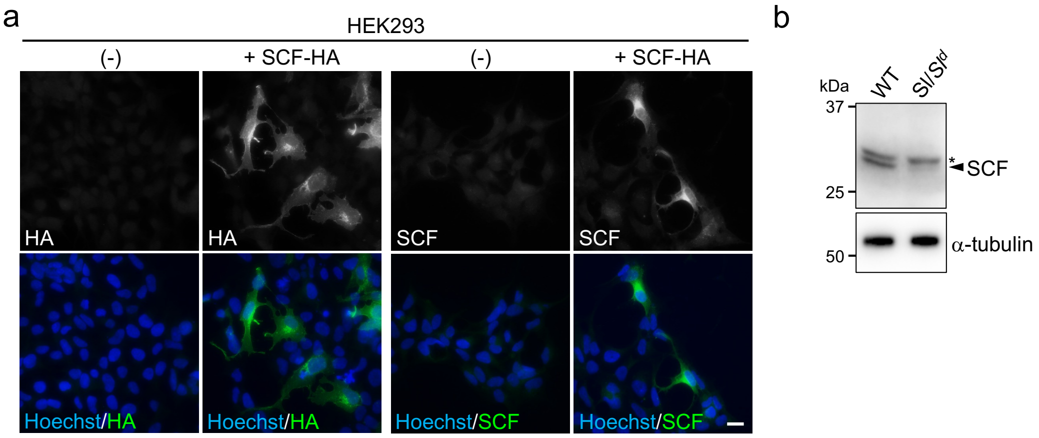


**Supplementary Fig. 15 | Characterization of rabbit anti-SCF polyclonal antibody.**

**a** Immunofluorescence staining of parental HEK293 cells and HEK293 cells transiently expressing SCF-HA using anti-HA or anti-SCF antibody. **b** Western blotting of testis lysates prepared from wild-type (WT) and *Sl*/*Sl*[d] mice at 6 weeks of age using anti-SCF and anti-α-tubulin antibodies. *Non-specific bands. Scale bar: 20 μm (**a**).

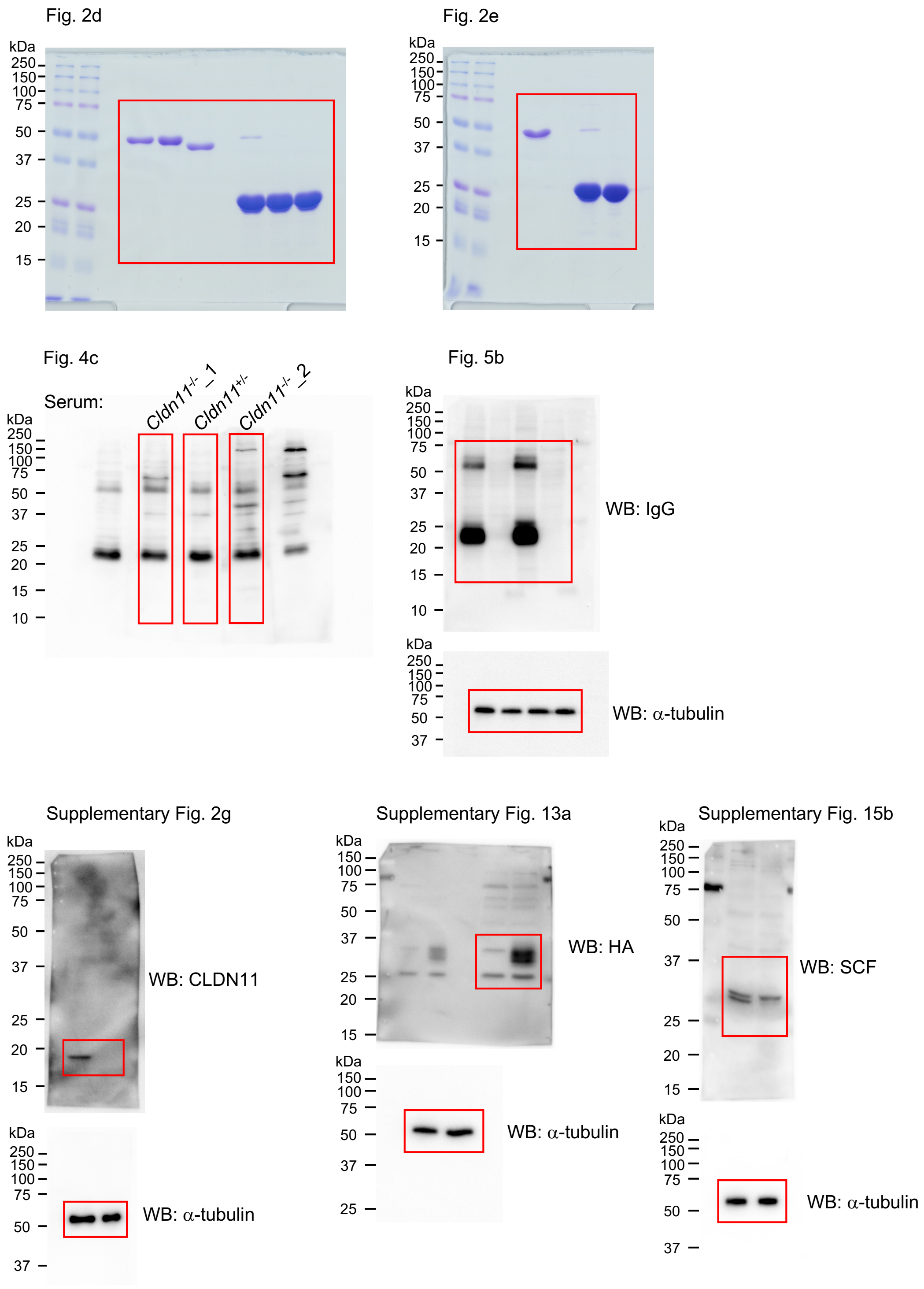


**Supplementary Fig. 16 | Uncropped gel and blot images.** Uncropped gel and blot images in Figs. 2d, 2e, 4c, 5b, Supplementary Figs. 2g, 13a, and 15b. Red squares indicate cropped regions.
