# Supplementary material for: Claudin-11 regulates immunological barrier formation and spermatogonial proliferation through stem cell factor
Source: Commun Biol. 2025 Jan 30;8:148. doi: 10.1038/s42003-025-07592-0 (PMC11782696; doi:10.1038/s42003-025-07592-0)
Supplement: Supplementary file 5 — Reporting Summary [file 42003_2025_7592_MOESM5_ESM.pdf]

nature portfolio

Corresponding author(s): Taichi Sugawara
Last updated by author(s): Jan 16, 2025

Reporting Summary

Nature Portfolio wishes to improve the reproducibility of the work that we publish. This form provides structure for consistency and transparency in reporting. For further information on Nature Portfolio policies, see our Editorial Policies and the Editorial Policy Checklist.

Statistics

For all statistical analyses, confirm that the following items are present in the figure legend, table legend, main text, or Methods section.

n/a	Confirmed	
☐	☒	The exact sample size (*n*) for each experimental group/condition, given as a discrete number and unit of measurement
☐	☒	A statement on whether measurements were taken from distinct samples or whether the same sample was measured repeatedly
☐	☒	The statistical test(s) used AND whether they are one- or two-sided<br>*Only common tests should be described solely by name; describe more complex techniques in the Methods section.*
☒	☐	A description of all covariates tested
☐	☒	A description of any assumptions or corrections, such as tests of normality and adjustment for multiple comparisons
☐	☒	A full description of the statistical parameters including central tendency (e.g. means) or other basic estimates (e.g. regression coefficient) AND variation (e.g. standard deviation) or associated estimates of uncertainty (e.g. confidence intervals)
☐	☒	For null hypothesis testing, the test statistic (e.g. *F*, *t*, *r*) with confidence intervals, effect sizes, degrees of freedom and *P* value noted<br>*Give P values as exact values whenever suitable.*
☒	☐	For Bayesian analysis, information on the choice of priors and Markov chain Monte Carlo settings
☒	☐	For hierarchical and complex designs, identification of the appropriate level for tests and full reporting of outcomes
☒	☐	Estimates of effect sizes (e.g. Cohen's *d*, Pearson's *r*), indicating how they were calculated

Our web collection on statistics for biologists contains articles on many of the points above.

Software and code

Policy information about availability of computer code

Data collection	Microscopy: cellSens Standard, FV31S-SW, and EMIP-SP
Data analysis	Image processing: Fiji/ImageJ (ver. 2.9.0/1.53t)<br>Statistical analysis: Microsoft Excel (ver. 16.78) and RStudio (ver. 2023.03.1+446)

For manuscripts utilizing custom algorithms or software that are central to the research but not yet described in published literature, software must be made available to editors and reviewers. We strongly encourage code deposition in a community repository (e.g. GitHub). See the Nature Portfolio guidelines for submitting code & software for further information.

Data

Policy information about availability of data

All manuscripts must include a data availability statement. This statement should provide the following information, where applicable:

- Accession codes, unique identifiers, or web links for publicly available datasets
- A description of any restrictions on data availability
- For clinical datasets or third party data, please ensure that the statement adheres to our policy

All data supporting the findings of this study are available within the paper and its Supplementary Information.

Human research participants

Policy information about studies involving human research participants and Sex and Gender in Research.

Reporting on sex and gender	N/A
Population characteristics	N/A
Recruitment	N/A
Ethics oversight	N/A

Note that full information on the approval of the study protocol must also be provided in the manuscript.

Field-specific reporting

Please select the one below that is the best fit for your research. If you are not sure, read the appropriate sections before making your selection.

☒ Life sciences ☐ Behavioural & social sciences ☐ Ecological, evolutionary & environmental sciences

For a reference copy of the document with all sections, see nature.com/documents/nr-reporting-summary-flat.pdf

Life sciences study design

All studies must disclose on these points even when the disclosure is negative.

Sample size	Sample sizes were determined based on previously published studies in the field.<br>Statistical methods were not used to predetermine sample size.
Data exclusions	N/A
Replication	Biological replicates for statistical analyses are described in figure legends and results.<br>All attempts at replication were successful.
Randomization	Animals were allocated into experimental groups based on the genotypes.
Blinding	Investigators were not blinded to group allocation during experiments.

Reporting for specific materials, systems and methods

We require information from authors about some types of materials, experimental systems and methods used in many studies. Here, indicate whether each material, system or method listed is relevant to your study. If you are not sure if a list item applies to your research, read the appropriate section before selecting a response.

Materials & experimental systems

n/a	Involved in the study	
☐	☒	Antibodies
☐	☒	Eukaryotic cell lines
☒	☐	Palaeontology and archaeology
☐	☒	Animals and other organisms
☒	☐	Clinical data
☒	☐	Dual use research of concern

Methods

n/a	Involved in the study	
☒	☐	ChIP-seq
☒	☐	Flow cytometry
☒	☐	MRI-based neuroimaging

Antibodies

Antibodies used	Rat monoclonal anti-ZO1 (1:1; clone: R26.4C, Developmental Studies Hybridoma Bank)<br>Mouse monoclonal anti-ZO1 (1:10; clone: T8-754, Itoh et al., J. Cell Biol., 115, 1449–1462, 1991)<br>Rat monoclonal anti-OCLN (1:1; clone: MOC37, Saitou et al., Eur. J. Cell Biol., 73, 222–231, 1997)<br>Rabbit polyclonal anti-OCLN (1:500; Saitou et al., Eur. J. Cell Biol., 73, 222–231, 1997)<br>Rabbit polyclonal anti-CLDN3 (1:300; catalog number: 34-1700, Thermo Fisher Scientific)<br>Rabbit polyclonal anti-CLDN5 (1:300; catalog number: 34-1600, Thermo Fisher Scientific)

	Rabbit polyclonal anti-CLDN11 (1:500 for immunofluorescence; Morita et al., J. Cell Biol., 145, 579–588, 1999)<br>Rabbit polyclonal anti-CLDN11 (1:1500 for western blotting; catalog number: ab53041, Abcam)<br>Rabbit polyclonal anti-JAM1 (1:300; catalog number: 36-1700, Thermo Fisher Scientific)<br>Rat monoclonal anti-CDH1 (1:500; catalog number: M108, Takara)<br>Rat monoclonal anti-NECTIN2 (1:300; catalog number: ab16912, Abcam)<br>Rabbit polyclonal anti-GJA1 (1:300; catalog number: C6219, Sigma–Aldrich)<br>Rabbit polyclonal anti-EZR (1:300; catalog number: sc-20773, Santa Cruz Biotechnology)<br>Mouse monoclonal anti-ATP1A1 (1:600; catalog number: NB300-146, Novus Biologicals)<br>Goat polyclonal anti-GFRA1 (1:300; catalog number: AF560, R&D Systems)<br>Rabbit polyclonal anti-PLZF (1:500; catalog number: HPA001499, Sigma–Aldrich)<br>Goat polyclonal anti-PLZF (1:300; catalog number: AF2944, R&D Systems)<br>Goat polyclonal anti-LIN28A (1:300; catalog number: AF3757, R&D Systems)<br>Goat polyclonal anti-KIT (1:300; catalog number: AF1356, R&D Systems)<br>Rabbit polyclonal anti-SCP3 (1:300; catalog number: ab15093, Abcam)<br>Rabbit polyclonal anti-VASA (1:500; catalog number: ab13840, Abcam)<br>Rabbit monoclonal anti-WT1 (1:500; catalog number: ab89901, Abcam)<br>Rabbit polyclonal anti-SCF (1:100 for immunofluorescence or 1:1500 for western blotting; catalog number: ab64677, Abcam)<br>Rabbit polyclonal anti-Ki67 (1:300; catalog number: ab15580, Abcam)<br>Rat monoclonal anti-CD45 (1:300; catalog number: 103101, BioLegend)<br>Rabbit polyclonal anti-HA (1:500 for immunofluorescence or 1:1500 for western blotting; catalog number: 561, MBL)<br>Mouse monoclonal anti-alpha-tubulin (1:2000; catalog number: T6199, Sigma–Aldrich)<br>Alexa Fluor 488-conjugated donkey anti-rabbit IgG (1:400; catalog number: ab150073, Abcam)<br>Alexa Fluor 555-conjugated donkey anti-rabbit IgG (1:400; catalog number: ab150074, Abcam)<br>Alexa Fluor 488-conjugated donkey anti-mouse IgG (1:400; catalog number: ab150105, Abcam)<br>Alexa Fluor 594-conjugated donkey anti-mouse IgG (1:400; catalog number: ab150108, Abcam)<br>Alexa Fluor 488-conjugated donkey anti-rat IgG (1:400; catalog number: ab150153, Abcam)<br>Alexa Fluor 594-conjugated donkey anti-rat IgG (1:400; catalog number: ab150156, Abcam)<br>Alexa Fluor 488-conjugated donkey anti-goat IgG (1:400; catalog number: ab150129, Abcam)<br>Alexa Fluor 594-conjugated donkey anti-goat IgG (1:400; catalog number: ab150132, Abcam)<br>Horseradish peroxidase (HRP)-conjugated goat anti-rabbit IgG (1:1500; catalog number: ab6721, Abcam)<br>HRP-conjugated goat anti-mouse IgG (1:1500; catalog number: ab6789, Abcam)
Validation	All antibodies were validated in previous papers or by manufacturers.

Eukaryotic cell lines

Policy information about cell lines and Sex and Gender in Research

Cell line source(s)	L cells (mouse fibroblasts), MDCK II cells (canine kidney epithelial cells), and HEK293 cells (human embryonic kidney cells) were provided by Masatoshi Takeichi (RIKEN Center for Biosystems Dynamics Research, Kobe, Japan), Masayuki Murata (Tokyo Institute of Technology, Yokohama, Japan), and Akira Tsuji (Kanazawa University, Kanazawa, Japan), respectively.
Authentication	None of the cell lines were authenticated.
Mycoplasma contamination	The cell lines were not tested for mycoplasma contamination.
Commonly misidentified lines (See ICLAC register)	N/A

Animals and other research organisms

Policy information about studies involving animals; ARRIVE guidelines recommended for reporting animal research, and Sex and Gender in Research

Laboratory animals	Wild-type and genetically modified male mice on a C57BL/6J background were analyzed at postnatal day 10, 20, 30, or 10–12 weeks of age.<br>WBB6F1-+/+ and WBB6F1-Sl/Sld male mice purchased from Japan SLC, Inc. were analyzed at 6 weeks of age.
Wild animals	The study did not involve wild animals.
Reporting on sex	Our findings apply to only male mice.<br>Female mice were not analyzed in this study.
Field-collected samples	The study did not involve samples collected from the field.
Ethics oversight	The Animal Care and Use Committee of Kumamoto University approved the study protocol.

Note that full information on the approval of the study protocol must also be provided in the manuscript.
