# Supplementary material for: Claudin-11 regulates immunological barrier formation and spermatogonial proliferation through stem cell factor
Source: Commun Biol. 2025 Jan 30;8:148. doi: 10.1038/s42003-025-07592-0 (PMC11782696; doi:10.1038/s42003-025-07592-0)
Supplement: Supplementary file 1 — Peer review file [file 42003_2025_7592_MOESM1_ESM.pdf]

nature portfolio

Peer Review File

Claudin-11 regulates immunological barrier formation and spermatogonial proliferation through stem cell factor

Corresponding Author: Dr Taichi Sugawara

This file contains all reviewer reports in order by version, followed by all author rebuttals in order by version.

Version 0:

Reviewer comments:

Reviewer #1

(Remarks to the Author)
In this manuscript, the roles of CLDN11 in Sertoli cell polarization, localization of SCF at the basal compartment of seminiferous tubules, and prevention of autoantibody production are being reported. Although the implication of CLDN11 in the establishment of the blood testis barrier has been well documented previously, current research better defines how CLDN11 contributes to the establishment of this immunological barrier. Overall, the manuscript is well written and very interesting. However, I have several comments needing to be considered:

Major comments:
1. Regarding the presentations of box plots in supplemental figures 5a, b, figure 1d, e, f, and 7d what are representing the dots in the figure? Technical replicates for each mice? If so, different symbols should be used for different mice to highlight the number of biological replicates. Also, the statistics should be performed on the biological replicates (average of technical replicates per mice) and not on total technical replicates. Results should be re-interpreted accordingly.
2. For supplemental figures 4, 7b, 10a and figures 1a and 5a, individual data points should be presented in the figures.
3. Throughout the entire manuscript comparisons are made between full Cldn11 knockouts (-/-) and heterozygotes (-/+). Moreover, the heterozygotes (Cldn11-/+) are used as control mice, whereas wild type mice (Cldn11+/+) should have been used. Current data does not allow to make a full assessment of the consequences of Cldn11 inactivation. For instance, are there phenotypic differences between Cldn11-/+ and Cldn11+/+? This should be supported by immunofluorescence data.
4. Regarding the impact of Cldn11 knockout on localization of TJ-associated proteins (Supplementary Fig. 8) why comparisons were made between Cldn11+/+ and Cldn11-/- and not between Cldn11+/- and Cdln11-/-?
5. Sertoli cell barrier function of Cldn-/- should also be analyzed and presented as in Fig 3b. Data from Fig. 4d should also be presented for Cldn-/-.
6. Regarding the results section on Rag2 knockout on page 11, are the Rag2-/- also Jak3-/-? If so, this should be presented in figure 5. In figure 5c,d,e why not show the Cldn+/-,Rag-/- data? Is there a difference compared to Cldn+/+,Rag2-/-?
7. Regarding the results presented in figure 7c: although suggesting that SCF induces the proliferation of differentiating Spg, would it be possible to support this finding with cross-sections IF from the in vivo transplanted SCF-soaked beads in addition to whole-mount immunofluorescence? This may be more convincing.
8. In the discussion section, lines 403-416, authors report the discrepancies between the research from Gow et al. 1999 and their findings. They highlight that autoantibodies were not detected in all sera from Cldn11-/- mice. Is it possible to elaborate on the cause of such difference between Cldn11 knockout mice?

Minor comments:
1. The text presenting data in figure 1d and e (lines 130-139) does not consider or explain the normalization of results to WT1 positive Sertoli cells.
2. Line 450, please remove reference to Fig. 7e.

Reviewer #2

(Remarks to the Author)
Please see the pdf attachment of critique.

Reviewer #3

(Remarks to the Author)
To address whether Sertoli cell TJs (SCTJs) do indeed form immunological barriers, the authors analyzed male mice lacking claudin-11 (Cldn11), which encodes a SCTJ component, and found autoantibodies against antigens of spermatocytes/spermatids in their sera. However, defective spermatogenesis was not restored in Cldn11-deficient mice on a genetic background mimicking a severely impaired adaptive immune system, leading to the conclusion that defective spermatogenesis is not caused by autoimmune responses against spermatogenic cells. They further observed that Cldn11 knockout impaired Sertoli cell polarization and localization of stem cell factor (SCF) (a key molecule for maintaining differentiating spermatogonia) to the basal compartment of seminiferous tubule. The authors propose that CLDN11 creates a microenvironment for SCF-mediated spermatogonial proliferation at the basal compartment via Sertoli cell polarization.
The manuscript has been written clearly in all sections with a nice review of the previous work in the field in the introduction and discussion. The authors also independently confirmed the earlier findings, in this work, such as the characterization of Sl/Sld mice and the conclusions are drawn based on valid findings. It is noted that the manuscript has been published in the preprint server bioRxiv, which is reasonable. The findings presented in this manuscript are of relevance for the scientific community in the field and for the broader audience.

Comments:
1. The authors can provide more details on the bead preparation and transplantation of Affi-Gel beads (which is different than Uchida et al 2016), such as the concentration of the beads, volume injected per testis and sites injected per testis. A Hamilton syringe used? If so, what size syringe was used (volume)?
2. On page 10, it says “antigens are present in 9 of 20 sera from Cldn11-/- mice compared with 2 of 26 sera from control mice. But wondering whether the value should be actually 4 out of 26 mice for control mice (2/26 as explained in lines 218-219 and 2/26 fluorescence signals as explained on lines 224-225).

Open Access This Peer Review File is licensed under a Creative Commons Attribution 4.0 International License, which permits use, sharing, adaptation, distribution and reproduction in any medium or format, as long as you give appropriate credit to the original author(s) and the source, provide a link to the Creative Commons license, and indicate if changes were made.
In cases where reviewers are anonymous, credit should be given to 'Anonymous Referee' and the source.
The images or other third party material in this Peer Review File are included in the article’s Creative Commons license, unless indicated otherwise in a credit line to the material. If material is not included in the article’s Creative Commons license and your intended use is not permitted by statutory regulation or exceeds the permitted use, you will need to obtain permission directly from the copyright holder.
To view a copy of this license, visit https://creativecommons.org/licenses/by/4.0/

Rebuttal 1

Reviewer #1 (Remarks to the Author):

In this manuscript, the roles of CLDN11 in Sertoli cell polarization, localization of SCF at the basal compartment of seminiferous tubules, and prevention of autoantibody production are being reported. Although the implication of CLDN11 in the establishment of the blood testis barrier has been well documented previously, current research better defines how CLDN11 contributes to the establishment of this immunological barrier. Overall, the manuscript is well written and very interesting. However, I have several comments needing to be considered:

Response
We are glad that the reviewer was interested in our studies. We responded to the comments by the reviewer below in a point-by-point manner.

Major comments:
1. Regarding the presentations of box plots in supplemental figures 5a, b, figure 1d, e, f, and 7d what are representing the dots in the figure? Technical replicates for each mice? If so, different symbols should be used for different mice to highlight the number of biological replicates. Also, the statistics should be performed on the biological replicates (average of technical replicates per mice) and not on total technical replicates. Results should be re-interpreted accordingly.

Response
In Supplementary Fig. 5a, b, Figs. 1d, e, f and 7d, the dots in box-and-whisker plots indicate outliers in technical replicates. In the revised figure, we have shown mean ± SD with red dots indicating biological replicates of mice on the graph. Accordingly, the statistical analyses were performed on the biological replicates. Our conclusions were not altered after the statistical analyses.

2. For supplemental figures 4, 7b, 10a and figures 1a and 5a, individual data points should be presented in the figures.

Response
In accordance with the reviewer's suggestion, we have shown individual data points as red dots indicating biological replicates of mice in the Supplementary Figs. 4, 7b, 10a, Figs. 1a and 5a in the revised manuscript.

3. Throughout the entire manuscript comparisons are made between full Cldn11 knockouts (-/-) and heterozygotes (-/+). Moreover, the heterozygotes (Cldn11-/+) are used as control mice, whereas wild type mice (Cldn11+/+) should have been used. Current data does not allow to make a full assessment of the consequences of Cldn11 inactivation. For instance, are there phenotypic differences between Cldn11-/+ and Cldn11+/+? This should be supported by immunofluorescence data.

Response

As the reviewer pointed out, comparison of phenotypes in the testis between *Cldn11*$^{+/+}$ and *Cldn11*$^{+/-}$ mice is necessary. Therefore, we investigated testicular histology and accumulation of spermatozoa in the cauda epididymides in *Cldn11*$^{+/+}$ and *Cldn11*$^{+/-}$ mice by HE staining (Supplementary Fig. 2a, b). Furthermore, we examined localization of TJ-associated proteins (CLDN11, OCLN, and ZO1) by immunohistochemistry of frozen testis sections from *Cldn11*$^{+/+}$ and *Cldn11*$^{+/-}$ mice (Supplementary Fig. 2c–e). These analyses demonstrate that there are no notable phenotypic differences between *Cldn11*$^{+/+}$ and *Cldn11*$^{+/-}$ mice. This conclusion is consistent with the previous study (Gow et al., Cell, 1999: Ref. 17). Thus, in this study, we used *Cldn11*$^{+/+}$ and *Cldn11*$^{+/-}$ mice as controls for *Cldn11*$^{-/-}$ mice. We have presented the data in the revised manuscript (Supplementary Fig. 2a–e) and described this point in the Results section (line 118–124).

4. Regarding the impact of Cldn11 knockout on localization of TJ-associated proteins (Supplementary Fig. 8) why comparisons were made between Cldn11+/+ and Cldn11-/- and not between Cldn11+/- and Cdln11-/-?

Response

As mentioned above (Response to the major comment 3), we could not find remarkable phenotypic differences between *Cldn11*$^{+/+}$ and *Cldn11*$^{+/-}$ mice and thus concluded that both *Cldn11*$^{+/+}$ and *Cldn11*$^{+/-}$ mice are appropriate for controls of *Cldn11*$^{-/-}$ mice.

5. Sertoli cell barrier function of Cldn-/- should also be analyzed and presented as in Fig 3b. Data from Fig. 4d should also be presented for Cldn-/-.

Response

In accordance with the reviewer's suggestion, we have added immunofluorescence image data of tracer experiments using *Cldn11*$^{-/-}$ mouse testes in Fig.3b in the revised manuscript.

Regarding Fig. 4d, frozen sections prepared from testes fixed with 4% PFA were used for immunohistochemistry particularly to detect IgG proteins as described in the Methods section in the original manuscript (line 561–564). Unfortunately, however, it was difficult to detect TJ-associated proteins other than CLDN11 in this experimental condition. It is reasonable that CLDN11 is lost in *Cldn11*$^{-/-}$ mice. Thus, in Fig. 4d, we would like to present the result of tracer experiments using only *Cldn11*$^{+/-}$ mouse testes. We believe that the immunofluorescence image data are sufficient to state that IgG proteins cannot penetrate into seminiferous tubules across CLDN11-positive SCTJs, and Sertoli cell barrier function against IgG proteins is lost in *Cldn11*$^{-/-}$ mice as shown in Fig. 4e, f.

6. Regarding the results section on Rag2 knockout on page 11, are the Rag2-/- also Jak3-/-? If so, this should be presented in figure 5. In figure 5c,d,e why not show the Cldn+/-,Rag-/- data? Is there a difference compared to Cldn+/+,Rag2-/-?

Response

All mice used in Fig. 5 have wild-type alleles of *Jak3*. We have clearly described this point in the Results (line 266–267) and Methods (line 484–489) in the revised manuscript. Regarding the genotype of *Cldn11* in Fig. 5c–e, as mentioned above (Response to the major comment 3), *Cldn11*$^{+/+}$ and *Cldn11*$^{+/-}$ mice can be used as controls for *Cldn11*$^{-/-}$ mice. We could not find notable histological differences in testes between *Cldn11*$^{+/+}$/*Rag*$^{-/-}$ and *Cldn11*$^{+/-}$/*Rag2*$^{-/-}$ mice.

7. Regarding the results presented in figure 7c: although suggesting that SCF induces the proliferation of differentiating Spg, would it be possible to support this finding with cross-sections IF from the in vivo transplanted SCF-soaked beads in addition to whole-mount immunofluorescence? This may be more convincing.

Response

In accordance with the reviewer's valuable comment, we have presented immunohistochemical data using testis sections prepared from *Cldn11*$^{-/-}$ mouse testes into which BSA or SCF-soaked beads were transplanted in Supplementary Fig. 14. We observed KIT-positive differentiating spermatogonia in seminiferous tubules close to SCF-soaked beads, but not BSA-soaked beads, supporting our results obtained by whole-mount immunofluorescence (Fig. 7c, d). We have described this result in the Results section in the revised manuscript (line 349–351).

8. In the discussion section, lines 403-416, authors report the discrepancies between the

research from Gow et al. 1999 and their findings. They highlight that autoantibodies were not detected in all sera from Cldn11-/- mice. Is it possible to elaborate on the cause of such difference between Cldn11 knockout mice?

Response

As described in the original manuscript, we detected autoantibodies in 9 of 20 sera from *Cldn11*$^{-/-}$ mice by immunohistochemistry of wild-type testis sections, indicating that autoantibodies could not be detected in all sera from *Cldn11*$^{-/-}$ mice. Gow *et al*. reported that they examined four sera from *Cldn11*$^{-/-}$ mice by immunohistochemistry. Thus, it is possible that none of the sera they analyzed reacted to testicular antigens. It should also be noted that the titer and amount of autoantibodies may differ among samples, and the differences may influence the experimental results. In addition, our methods for immunohistochemistry might be different from theirs. We performed heat-induced antigen retrieval using 20 mM Tris-HCl buffer (pH 9.0) at 95 °C for 15 min after deparaffinization of testis sections. Meanwhile, we are wondering whether Gow *et al*. performed such treatment for immunohistochemistry because they didn't describe the point (Gow et al., Cell, 1999: Ref. 17). We have discussed this point in the Discussion section in the revised manuscript (line 424–430).

Minor comments:

1. The text presenting data in figure 1d and e (lines 130-139) does not consider or explain the normalization of results to WT1 positive Sertoli cells.

Response

We thank the reviewer's suggestion. In the figure legend of the original manuscript, we explained that the number of KIT^{+}, GFRA1^{+}, PLZF^{+}, or LIN28A^{+} cells was normalized to the number of WT^{+} Sertoli cells. In addition, we have clearly described this point in the Results section of the revised manuscripts (line 137–141 and 144–149).

2. Line 450, please remove reference to Fig. 7e.

Response

In accordance with the reviewer's comment, we have removed the reference to Fig. 7e in the revised manuscript (line 469).

<u>Reviewer #2 (Remarks to the Author):</u>

This study presents clear evidence that the destruction of the blood-testis barrier (BTB), caused by the Claudin- 11 (Cldn11) null mutation, leads to the leakage of autoantigens and the production of autoantibodies. The experimental paradigm is solid and approaches are well designed and executed. Data interpretations are conservative and generally support authors' conclusions. Text is written straightforwardly and easy to follow the authors' logics. Concerns can be raised in that key findings are mostly qualitative and that the expression style to describe data and interpretations needs to be modified to make them more acceptable to a larger range of audience. Considering that the tasks that the authors took are a challenging one, this study is well appreciated overall.

Response
We are glad that the reviewer appreciated the significance of our studies and acknowledge valuable comments to our studies. We responded to the reviewer's comments one by one and revised the original manuscript to make our conclusions widely acceptable.

General Issues:
1. The use of Rag2 mice is clever in addressing the contribution of immunological reaction to spermatogenic defects in Cldn11-knockout (KO) mice. Multiple clarifications are required for this experiment.
a) The process of mutant mice production (i.e., how strains were crossed in how many generations) needs to be detailed.

Response
We obtained *Rag2*$^{-/-}$/*Jak3*$^{-/-}$ mice on a C57BL/6 background from the Center for Animal Resources and Development (Kumamoto University, Japan; ID576)[41]. To generate *Cldn11*$^{-/-}$/*Rag2*$^{-/-}$ mice with wild-type alleles of *Jak3*, *Cldn11*$^{+/-}$/(*Rag2*$^{+/-}$ or *Rag2*$^{-/-}$)/(*Jak3*$^{+/+}$ or *Jak3*$^{+/-}$) male mice were mated with *Cldn11*$^{-/-}$/(*Rag2*$^{+/-}$ or *Rag2*$^{-/-}$)/(*Jak3*$^{+/+}$ or *Jak3*$^{+/-}$) female mice. To generate (*Cldn11*$^{+/-}$ or *Cldn11*$^{-/-}$)/(*Rag2*$^{+/-}$ or *Rag2*$^{-/-}$)/(*Jak3*$^{+/+}$ or *Jak3*$^{+/-}$) mice within at least 3 generations, *Cldn11*$^{+/+}$/*Rag2*$^{-/-}$/*Jak3*$^{-/-}$ male mice were mated with *Cldn11*$^{-/-}$/*Rag2*$^{+/+}$/*Jak3*$^{+/+}$ female mice, and the obtained pups were mated with one another. We have described this point in the Method section in the revised manuscript (line 484–489).

b) Although the authors indicate this immunological mutant model simply Cldn11-/-;Rag2-/- in text, these mice also lack Jak3, which can be a compounding factor for data interpretation. The authors need to explain how the involvement of Jak3-/- could affect the experimental

paradigm and the overall physiology of mutant mice. Then, a justification needs to be presented to support the authors' data interpretation, which does not consider the Jak3 deletion.

Response

As mentioned above (Response to the General Issue 1a), in this study, we used *Cldn11*$^{-/-}$/*Rag2*$^{-/-}$ male mice with wild-type alleles of *Jak3*. We have clarified this point in the Result section (line 266–267) and the Methods section (line 484–489) in the revised manuscript.

c) The authors found that crossing with Rag2 mice did not improve the spermatogenesis in Cldn11-KO mice, leading to their conclusion that the autoimmune rejection is not a cause of spermatogenic defects. This is a very strong statement. It is more logical that the lack of B cells and T cells did not (or was not sufficiently strong to) "rescue" the spermatogenic phenotype of Cldn11-KO mice. Disruption of BTBs, as the authors clarified in this study, causes a wide-spread and profound destruction in the "structural integrity" of the seminiferous epithelium. The results of BTB loss are thus multifaceted, and immunological reaction is merely one of them. When the loss of structural integrity is so devastating that all meiotic and haploid cells are lost, immunological events are likely masked, even if they could be a part cause and contribute to spermatogenic defects. After all, it is not possible to induce only immunological reactions without altering other anatomical and functional perspectives of the seminiferous epithelium. Thus, it is thought to be more reasonable to state that Rag2 phenotype "failed to rescue" the spermatogenic defects observed in Cldn11-KO. Inclusion of this discussion should make the authors' conclusion more widely acceptable.

Response

As the reviewer pointed out, our conclusion that autoimmune responses are not a cause of spermatogenic defects in *Cldn11*$^{-/-}$ mice (line 30–31, 101–103, and 252–254 in the original manuscript) is a very strong statement because autoimmune responses via other factors including innate immunity and/or the complement system may be involved in spermatogenic defects in *Cldn11*$^{-/-}$ mice. Meanwhile, however, it is safe to state that humoral and cellular immune responses associated with B- and T-cell function are not a cause of spermatogenic defects at least in the *Cldn11*$^{-/-}$ mouse model because additional deletion of *Rag2*, which is essential for adaptive immune responses, cannot rescue defective spermatogenic phenotypes in *Cldn11*$^{-/-}$ mice at all. We have changed the original statement in the revised manuscript (line 30–31, 103–104, and 262–264).

As the reviewer pointed out, we should also note that some immunological events in

Cldn11$^{-/-}$ mice are likely masked due to profound effects of SCTJ disorganization by *Cldn11* knockout on structural integrity of seminiferous epithelia and differentiation of spermatocytes/spermatids even if the immunological reactions could partially contribute to spermatogenic defects. We have discussed this point in the Discussion section in the revised manuscript (line 414–417).

2. The authors used the terminology "regulate" quite frequently and liberally with a broad range of implications, and revising some of them may be beneficial. For instance, Cldn11 may regulate SCF localization, but it merely "contributes to supporting" spermatogonial proliferation and differentiation via SCF, rather than directly "controlling" them. It is suggested that the authors consider altering "regulate" to other words (contribute, promote, maintain, etc.) on Lines 273, 302, 305, 339, 417, and 454.

Response

In accordance with the reviewer's comment, we have changed the terminology "regulate" on line 273, 302, 305, 339, 417, and 454 in the original manuscript to other words ("contribute", "maintain", or "promote") in the revised manuscript (line 283, 312, 315, 351, 436, and 473).

Specific Issues:

1. DDX4 is the proper terminology for VASA.

Response

In accordance with the reviewer' suggestion, we have used "DDX4" in the revised manuscript instead of "VASA".

2. Is Supplemental Fig. 1 cited from Ref. 33? If so, it needs to be clarified in the legend.

Response

Based on single-cell RNA-seq data obtained from https://doi.org/10.17632/kxd5f8vpt4.1 (Ref. 33), we generated Supplementary Fig. 1. In accordance with the reviewer's suggestion, we have clarified the reference in the legend for Supplementary Fig. 1 in the revised manuscript.

3. Clarify the ages or age range of experimental mice.

Response

Unless specified otherwise, male mice at 10–12 weeks of age were analyzed in this study as

described on line 493 in the Methods section in the revised manuscript.

4. Quantitative data (Fig. 1) were normalized using the number of WT1+ Sertoli cells. This is logical because KO mice lack spermatogenesis that makes seminiferous tubules shorter and remaining cells concentrated in a short distance/small area. Perhaps, only the caveat is that this approach, which has been widely used throughout our research field, is valid only when Sertoli cells are not overly affected. The authors analyzed apoptosis in mutant testes. Were there any apoptosis noted on Sertoli cells?

Response

We examined whether spermatogenic cells underwent apoptosis in *Cldn11*$^{-/-}$ mice using TUNEL staining assay (Fig. 1f, g). In addition, we performed immunohistochemistry of testis sections from *Cldn11*$^{-/-}$ mice for WT1 with TUNEL staining assay and could not detect TUNEL signals in WT1^{+} nuclei as shown in Supplementary Fig. 6 in the original manuscript. This suggests that Sertoli cells don't undergo apoptosis and are not lost by *Cldn11* knockout. Therefore, we concluded that normalization using the number of WT1^{+} Sertoli cells in quantitative data (Fig. 1d, e and Supplementary Fig. 5a, b) was valid.

5. Experiments regarding autoantigens and autoantibodies need more details.

a) It should be informative to describe the methodology of autoantigen detection independently from other immunostatining procedures. Sera were used and the authors described how sera were obtained. But, how they were used for the staining procedure is unclear. Were the sera concentrated? What was the volume of blood acquired and what was the volume of sera obtained? How much of the serum was actually used for each staining experiment? The tighter of autoantibodies may well differ from sample to sample and depending on genotypes, which may well influence the staining results; yet, it is not measurable. Therefore, some more details of the protocols for autoantigen and autoantibody detections need to be presented. Can the relative amount of autoantibodies be "estimated" from Western blot results? As such, data interpretation may need to consider the variation in autoantibody amount and tighter.

Response

We collected 400–600 μl blood from the mouse heart and finally obtained 100–200 μl sera. Basically, methods of immunohistochemistry and western blotting for autoantigen detection were same with other methods using primary antibodies. Without concentration of sera, we directly used sera as primary antibodies (diluted 1:300 for immunohistochemistry; 1:500 for

western blotting). We have described the detailed methodology of autoantigen detection in the Methods section in the revised manuscript (line 566, 598, and 683).

As the reviewer pointed out, the titer of autoantibodies may differ among samples. In western blotting, we might be able to discuss the kind of autoantibodies against testicular antigens in terms of the number of bands which are not detected in control. However, it might be difficult to estimate the relative amount of autoantibodies because the chemiluminescence signal intensity of a band in western blotting depends on both the titer and amount of autoantibodies. When the titer and/or amount of autoantibodies is quite low, immunohistochemistry and western blotting do not allow us to detect them in sera from control mice and even *Cldn11*$^{-/-}$ mice. Thus, it should be noted that the titer and amount of autoantibodies may differ among samples, and the differences may influence the experimental results. We have described this interpretation regarding autoantibody detection in the Discussion section in the revised manuscript (line 427–430).

b) In Fig. 4, autoantigens seem to be preferentially detected in the nuclei. Do the authors have insights into this observation?

Response

In general, meiotic and postmeiotic male germ cells are thought to synthesize novel testis-specific antigens with high immunogenicity during the differentiation. However, it appears to be largely unknown what such autoantigens are, and where those are located in the germ cells. A study by another group may support our findings that antigens in the nuclei of spermatocytes/spermatids can be preferentially recognized as autoantigens. Meng et al. reported that SCTJ structures were disrupted in mice with Sertoli cell-specific ablation of the androgen receptor, and two of three sera from the mice included autoantibodies reacting to antigens associated with round and elongated spermatids (Meng et al., Biol. Reprod., 2011: Ref. 46). Their immunofluorescence images indicated that autoantibodies appeared to recognize nuclear antigens of the germ cells at the adluminal compartment, although the precise description regarding subcellular localization of the autoantigens was not shown. Thus, we would like to avoid this discussion in the revised manuscript. Identification of molecules recognized as autoantigens in spermatocytes/spermatids in *Cldn11*$^{-/-}$ mice might be useful to reveal the whole picture of CLDN11-mediated immunological barrier function in the testis.

c) Serum #1 seems to react preferentially the antigens in spermatocytes while Serum #2 spermatids. Does Serum #1 not detect spermatids at all? Likewise, how about Serum #2 on

spermatocyte? In this regard, presentation of staining results using normal serum should be informative to see which cell types and which cell structures are stained with normal serum.

Response

Serum #1 seems to react antigens in SCP3-negative round spermatids as well as antigens in SCP3-positive spermatocytes, while Serum #2 preferentially reacts to antigens in the PNA-positive acrosome of spermatids. As shown in Fig. 4a, remarkable fluorescence signals were not detected in stage I–VI or IX–X seminiferous tubules by immunohistochemistry using control serum as described in the original manuscript (line 218–219). This suggests that control serum doesn't react to antigens of spermatogenic cells including spermatocytes/spermatids or Sertoli cells in seminiferous tubules.

d) Fig. 4b is better to have colors for SCP3 alone and PNA alone. In the current images, four black- and-white panels all look the same. The same color issue applies to Supplemental Fig. 9.

Response

We thank the reviewer's suggestion for improvement of the figure presentation. In Fig. 4b, we emphasized the words "Serum" with the green color and "SCP3"/"PNA" with the red color, respectively, to avoid the confusion. In addition, to keep the consistency regarding the presentation of multicolor fluorescence images in whole figures, we would like to show the SCP3 and PNA images with black and white colors.

6. There have not been reports showing that MDCK II cells express Cldn11; do the authors have the results otherwise? (if they have the evidence that Cldn11 is expressed in MDCK II cells, please present it.) As long as MDCK II cells do not express Cldn11, the data generated using this cell line are correlative or do not directly address the function of Cldn11 for cell polarization. Thus, such statements as those on Lines 328-329 are misleading. It could be useful to consider removing experiments using MDCK II cells, which would likely not overly affect this study.

Response

As the reviewer pointed out, there have not been reports showing that MDCK II cells express CLDN11. We also don't have the evidence for CLDN11 expression in MDCK II cells. In this study, we used MDCK II cells as a well-polarized epithelial cell model to show the localization of mouse SCF to the lateral membrane, but not the contribution of CLDN11 to epithelial

polarization in the cells. This result is valuable to support the idea that SCF preferentially accumulates at the basal, but not adluminal, compartment of seminiferous tubules. However, we agree that the description on line 328–329 might be misleading. Thus, to avoid the misunderstanding, we have described the results of experiments using MDCK II cells immediately after the description regarding SCF localization in busulfan-treated *Cldn11*$^{+/-}$ mice in the revised manuscript (line 332–336).

Reviewer #3 (Remarks to the Author):

To address whether Sertoli cell TJs (SCTJs) do indeed form immunological barriers, the authors analyzed male mice lacking claudin-11 (Cldn11), which encodes a SCTJ component, and found autoantibodies against antigens of spermatocytes/spermatids in their sera. However, defective spermatogenesis was not restored in Cldn11-deficient mice on a genetic background mimicking a severely impaired adaptive immune system, leading to the conclusion that defective spermatogenesis is not caused by autoimmune responses against spermatogenic cells. They further observed that Cldn11 knockout impaired Sertoli cell polarization and localization of stem cell factor (SCF) (a key molecule for maintaining differentiating spermatogonia) to the basal compartment of seminiferous tubule. The authors propose that CLDN11 creates a microenvironment for SCF-mediated spermatogonial proliferation at the basal compartment via Sertoli cell polarization.
The manuscript has been written clearly in all sections with a nice review of the previous work in the field in the introduction and discussion. The authors also independently confirmed the earlier findings, in this work, such as the characterization of Sl/Sld mice and the conclusions are drawn based on valid findings. It is noted that the manuscript has been published in the preprint server bioRxiv, which is reasonable. The findings presented in this manuscript are of relevance for the scientific community in the field and for the broader audience.

Response
We are delighted that the reviewer appreciated the value of our research. We responded to the reviewer's comments below in a point-by-point manner.

Comments:
1. The authors can provide more details on the bead preparation and transplantation of Affi-Gel beads (which is different than Uchida et al 2016), such as the concentration of the beads,

volume injected per testis and sites injected per testis. A Hamilton syringe used? If so, what size syringe was used (volume)?

Response

As the reviewer pointed out, our methods for the bead preparation and transplantation are different from methods in the previous study (Uchida et al., *Biochem. Biophys. Res. Commun.*, 2016: Ref. 44) in some points. We injected a volume of 10 μl of PBS containing 26.0 ± 9.0 (mean ± SD) beads labeled with CM-DiI into the central regions of *Cldn11*$^{-/-}$ mouse testes using 29G insulin syringe (#326666; BD), but not Hamilton syringe. We have added this method in the Methods section in the revised manuscript (line 648–650).

2. On page 10, it says "antigens are present in 9 of 20 sera from Cldn11-/- mice compared with 2 of 26 sera from control mice. But wondering whether the value should be actually 4 out of 26 mice for control mice (2/26 as explained in lines 218-219 and 2/26 fluorescence signals as explained on lines 224-225).

Response

The rest of 26 control sera as explained on line 218–219 in the original manuscript are 2 sera, which are the same as 2 of 26 control sera as explained on line 224–225.
